# With strength and breath, I laugh at death: a cross-sectional survey on involvement in recreational sport, resocialization, and well-dying perception among older adults

**DOI:** 10.3389/fpsyg.2026.1840367

**Published:** 2026-07-27

**Authors:** Jinge Wu, Qinghao Guan, Rob Law, Yifan Zuo

**Affiliations:** 1 Kyung Hee University, Yongin-si, Gyeonggi-do, Republic of Korea; 2 University of Zurich, Zurich, Switzerland; 3 University of Macau, Macao, China; 4 Shenzhen University, Shenzhen, China

**Keywords:** involvement in recreational sports, older adults, peer relationships, resocialization, well-dying perception

## Abstract

As the world’s population continues to age, it becomes increasingly important to help older adults make sense of what it means to die well. On the basis of the theory of successful aging, this study investigated the mechanisms that influence recreational sport involvement, resocialization, and well-dying perception among older adults. In addition, the mediating effect of peer relationships on well-dying perception was investigated. The results suggest that involvement in recreational sport can indirectly influence well-dying perception through resocialization, and peer relationships have a positive moderated mediating effect on the pathways of involvement in recreational activity, resocialization, and well-dying perception. These findings indicate that recreational sports play a significant role in promoting successful aging. They also provide theoretical support and practical insights for developing intervention strategies that advance successful aging and end-of-life education. Furthermore, this study highlights the positive influence of resocialization and peer relationships on older adults’ perceptions of a good death, offering new perspectives on the mechanisms underlying the formation of end-of-life awareness among the elderly.

## 1 Introduction

*It is not the end of the physical body that should worry us. Rather, our concern must be to live while we are alive.* ——Elisabeth Kübler-Ross

Global population aging has become increasingly pronounced, with many countries already entering or approaching aging societies (World Health Organization, 2024). In this context, scholars and policymakers’ focus has shifted from merely extending life expectancy to enhancing the final stage of life with greater dignity, tranquility, and psychological readiness. This concern is captured by the concept of well-dying perception, which refers to individuals’ cognition, attitudes, and psychological readiness regarding the end of life (Liew, 2022). Rather than representing solely a form of preparation for imminent death, well-dying perception reflects an active and autonomous orientation toward aging and the final stage of life. In practice, many older adults hope to die with dignity at the end of life and to make adequate preparations before that moment arrives (Gudat et al., 2019; Hyoungjae and Hoonsik, 2024). Prior studies suggest that involvement in recreational sports may contribute positively to older adults’ end-of-life experiences (Bonab and Parvaneh, 2022; Wang et al., 2025). However, how involvement in recreational sports shapes older adults’ well-dying perception and through what mechanisms this influence occurs remains insufficiently understood.

In recent years, a growing body of research has begun to examine well-dying perception among older adults. Existing studies have primarily investigated this issue from the perspectives of medical care and nursing services, thereby providing an important foundation for understanding older adults’ well-dying perception (Lee et al., 2022; Liew, 2022). Nevertheless, several limitations remain in the literature. First, most studies have focused mainly on factors such as end-of-life care and life satisfaction, while paying relatively limited attention to the formation of a well-dying perception from the perspectives of involvement in recreational sports and social factors. Second, although some studies have begun to explore the associations among leisure activities, social participation, and well-dying perception (Hu and Kim, 2022), the underlying mechanisms linking these concepts remain insufficiently explored. Third, existing research has tended to identify factors associated with well-dying perception among older adults, while paying limited attention to the boundary conditions under which these relationships may vary (Kim et al., 2024; Park and Han, 2025). Accordingly, the present study seeks to examine the factors that may moderate the determinants of older adults’ well-dying perception. As an important social resource influencing older adults’ social participation, peer relationships have been shown to contribute positively to social adaptation and psychological well-being in later life (Chen and Feeley, 2014). However, whether peer relationships shape the mechanism through which involvement in recreational sports influences well-dying perception among older adults remains unclear.

To bridge these gaps, the present study applies successful aging theory to develop a research model linking involvement in recreational sports, resocialization, peer relationships, and well-dying perception. Specifically, the study first investigates the effects of involvement in recreational sports on older adults’ resocialization and well-dying perception. It then examines whether resocialization serves as an internal mechanism linking involvement in recreational sports with well-dying perception. Finally, by considering the peer relationships that older adults may develop through sport participation, this study further explores the moderating role of peer relationships in these associations. These findings are expected to provide theoretical insights into how recreational sports and social resources may contribute to holistic well-being among older adults.

## 2 Literature review

### 2.1 Theoretical framework

The concept of successful aging was proposed by Rowe and Kahn (1987) and was further revised and refined in subsequent research. This theory suggests that optimal functioning in later life is reflected not only in a low probability of disease and disability but also in high levels of cognitive and physical functioning and active social engagement. Together, these three components constitute the core elements of successful aging (Rowe and Kahn, 1997). In later theoretical refinements, Cheng (2014) argued that these three dimensions do not exist in isolation; rather, they may be hierarchically organized or interrelated, jointly shaping older adults’ overall developmental status (Liffiton et al., 2012). From this perspective, the present study seeks to examine the potential relationships among different dimensions of successful aging. Specifically, involvement in recreational sports reflects older adults’ engagement in health-related physical behaviors; resocialization and peer relationships capture their active social participation; and well-dying perception represents their cognitive understanding of, and psychological preparedness for, the end of life. Accordingly, this study adopts successful aging theory as its theoretical lens to examine the relationships among physical functioning, social participation, and cognition, thereby extending the explanatory power of successful aging theory in understanding the formation of well-dying perception among older adults.

### 2.2 Involvement in recreational sport

Involvement in recreational sports is defined as individuals’ voluntary participation in various activities during their leisure time, primarily aimed at promoting physical health, enhancing psychological well-being, developing social skills, and improving overall life satisfaction (Hashemi et al., 2021). Within the framework of successful aging theory, a high level of physical functioning is an important component of successful aging (Rowe and Kahn, 1997). Thus, as an important means of maintaining physical health, involvement in recreational sports provides a key pathway through which older adults may achieve successful aging.

Prior research has shown that recreational sport activities can enhance older adults’ quality of life and positive psychological states, thereby reducing death anxiety to some extent (Bonab and Parvaneh, 2022; Wang et al., 2025). This effect may stem from the positive experiences that individuals gain through involvement in recreational sports, which may help them develop a stronger sense of meaning in life and shape their cognition and attitudes toward the end of life (Kasuga et al., 2021; Sinclair, 2011). In addition, for older adults after retirement, recreational sports are not merely a form of physical activity but also an important means of maintaining social interaction and reconstructing interpersonal networks (Zhou et al., 2024). Recreational sports may function as a form of social capital by helping individuals build social networks and develop new social identities (Sánchez-Santos et al., 2024). In this context, social capital refers to the interpersonal relationships, enhanced trust, and norms of reciprocity and sharing that individuals develop through participation in sport activities (Bhandari and Yasunobu, 2009). These relationship networks and forms of social capital generated through involvement in recreational sports may help older adults reintegrate into their social environments and reconstruct their social roles. Taken together, this study proposes the following hypotheses:

*H1*: Involvement in recreational physical activity has a positive effect on well-dying perception.

*H2*: Involvement in recreational sport has a positive impact on resocialization.

### 2.3 Resocialization

Resocialization refers to the process through which individuals are reintegrated into society (Lee et al., 2014). The concept was originally used to describe how individuals readapt to new social norms after entering institutional settings, such as prisons, the military, or psychiatric hospitals. Recently, however, the concept of resocialization has been widely applied across multiple fields, including workplace adaptation and aging research (Myung et al., 2020; Xu et al., 2024). In studies of older adults, resocialization refers to the process through which older adults adapt to new living environments by establishing new sociocultural relationships, interpersonal relationships, and social connections (Cao et al., 2022). This process is typically reflected in active social participation and interpersonal interaction (Kim, 2014).

According to successful aging theory, active social engagement is also an essential component of successful aging (Rowe and Kahn, 1997). After retirement, older adults often face weakened social roles and reduced social connections; therefore, they need to rebuild their social ties. In this context, resocialization can be regarded as an important mechanism that promotes active social participation among older adults. Previous research has shown that individuals with higher levels of resocialization tend to demonstrate stronger psychological adjustment, which may help them face the end of life with a more open and rational attitude and reduce uncertainty and anxiety about death (Carr and Khodyakov, 2007). Although few studies have directly tested resocialization as a mediating variable, a substantial body of literature suggests that involvement in recreational sports can influence individuals’ psychological states and behavioral outcomes by promoting social participation and enhancing social activity (Nakagawa and Hülür, 2020; Spaaij, 2012). Taken together, this study proposes the following hypotheses:

*H3*: Resocialization has a positive effect on well-dying perception.

*H4*: Resocialization mediates the relationship between recreational sport involvement and well-dying perception.

### 2.4 Well-dying perception

Well-dying perception is a multidimensional psychological and social-cognitive process (Lloyd et al., 2016). It refers to individuals’ cognition, attitudes, and preparedness regarding the end of life, including their acceptance of death (Liew, 2022). Among older adults, well-dying perception is often understood as an integrative process that encompasses understanding of end-of-life care, reflection on the meaning of life, and the reconstruction of life meaning (Oh, 2025). Previous research has found that a higher level of well-dying perception may help older adults face the end of life with a more positive attitude and, to some extent, increase the likelihood of achieving successful aging (Kong et al., 2015).

According to successful aging theory, successful aging involves not only the maintenance of physical functioning and active social engagement but also positive psychological adaptation to changes in later life (Rowe and Kahn, 1997). However, related research suggests that existing theoretical models of aging have generally paid limited attention to older adults’ cognition in the final stage of life, particularly the role of well-dying perception (Otto et al., 2022). If successful aging is understood solely in terms of health, functioning, and social participation while neglecting how individuals understand and prepare for death, its explanatory power across the full course of later life may be constrained. Therefore, by integrating successful aging theory with well-dying perception, this study extends the application of successful aging theory to end-of-life issues.

### 2.5 Peer relationships

Peer relationships refer to interpersonal relationships that develop through interactions within small groups, in which members form close connections based on shared interests and friendship (Shao and Kang, 2022). For older adults, peers are not merely social partners but also important sources of support for emotion regulation and social belonging (Inoue et al., 2020). Within the framework of successful aging theory, peer relationships can also be viewed as a social resource that promotes active social participation (Wang and Tian, 2024). However, such peer relationships may not directly influence well-dying perception; rather, they may exert an indirect influence by shaping individuals’ level of social participation (Juezan and Osorno, 2022).

In addition, previous research suggests that when individuals lack relational satisfaction in social contexts, their intrinsic motivation, behavioral persistence, and psychological adjustment may be inhibited (Ryan and Deci, 2024). This implies that a lack of positive peer relationships may weaken older adults’ willingness to participate in recreational sports, reduce the frequency of social interaction, and even intensify death anxiety (Vance, 2014). Conversely, close peer relationships may strengthen older adults’ social integration and enhance their well-dying perception (Chopik, 2017). Therefore, this study makes the following prediction:

*H5*: Peer relationships have a significant moderating and mediating effect on the relationship among involvement in recreational sports, resocialization, and well-dying perception.

**Figure 1:**
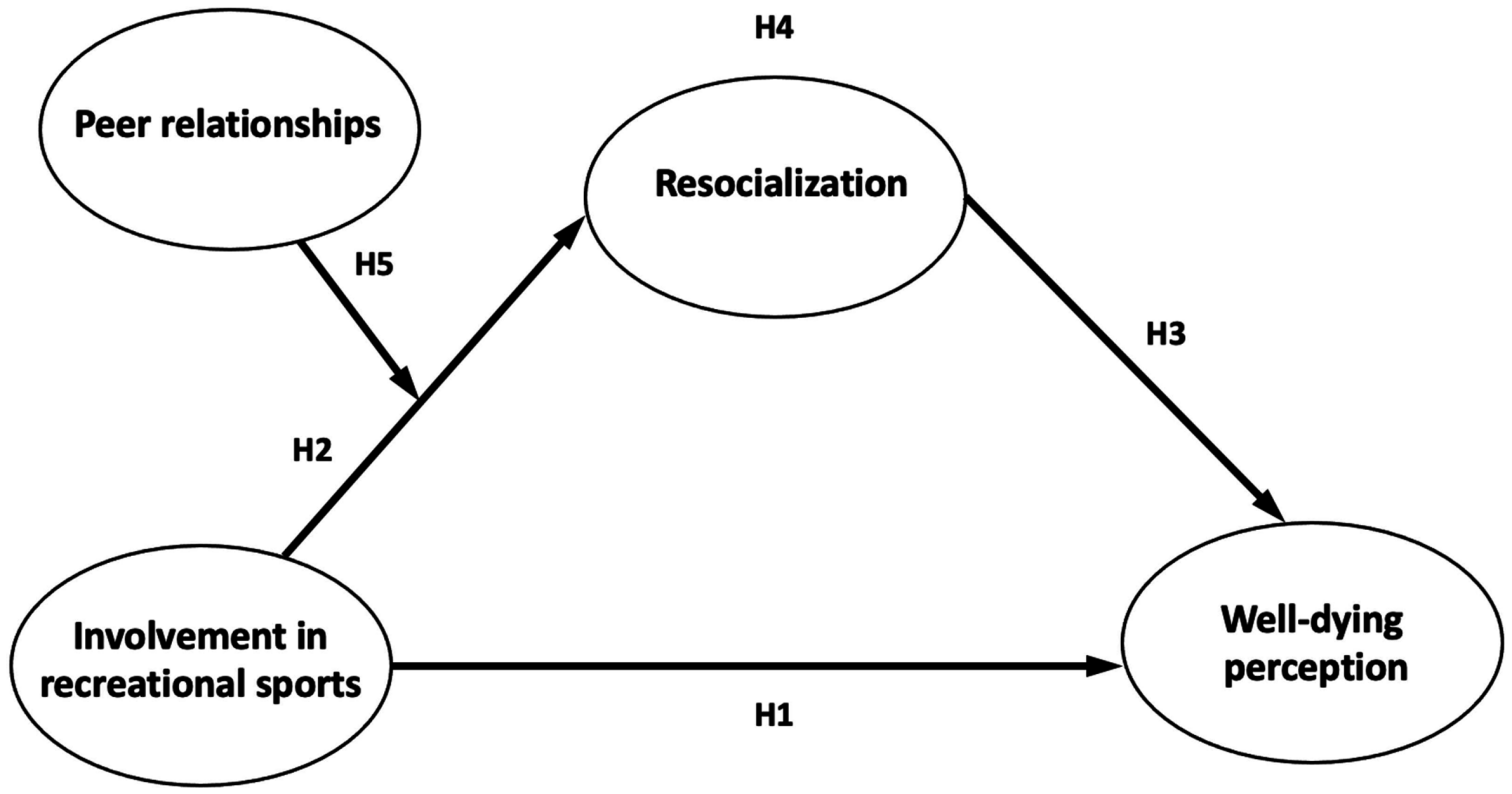
Proposed research model.

This study constructs a conceptual model to examine the relationships among recreational sports involvement, resocialization, peer relationships, and well-dying perception (Figure 1). The model integrates both mediating and moderating mechanisms to provide a comprehensive view of how physical, psychological, and social dimensions interact in shaping older adults’ perception of the end of life.

## 3 Research method

### 3.1 Data analysis method

This study used AMOS 24.0 and SPSS 26.0 for data processing and analysis. The analyses included descriptive statistics, confirmatory factor analysis (henceforth, CFA), reliability testing, correlation analysis, and tests for normal distribution, in order to establish the basic relationships among variables and to characterize the data distribution. Finally, Hayes’ PROCESS macro (Models 4 and 7) was utilized to test the research hypotheses, as it efficiently assesses mediation and moderated mediation effects while enhancing the robustness of results through bootstrapping.

### 3.2 Data collection

This study relied on a questionnaire that was distributed through social media platforms and employed non-probability sampling for questionnaire surveys from December 2024 to February 2025 in several provinces in China, including Shanghai, Beijing, Shaanxi, Shandong, Liaoning, and Henan. Considering the research context and target population, participants were primarily recruited from older adults who regularly engage in recreational sports, including members of senior sports clubs and community-based activity groups. Prior to the main survey, a pilot study was conducted to assess the clarity and feasibility of the questionnaire items and research variables. An electronic informed consent form was provided at the beginning of the questionnaire to obtain participants’ consent. Simultaneously, the questionnaire’s first page informed participants that this survey is anonymous and solely for research purposes. The survey targeted adults aged 50 and above. A total of 340 questionnaires were collected, of which 27 invalid responses were excluded, resulting in a final sample of 313. In addition, the study received review and approval from the Academic Ethics Committee (PN-202400175). Table 1 presents the demographic characteristics of the respondents.

**Table 1:** Background of participants.

Statical variable	Category	Frequency	Percentage (%)
Gender	Male	157	50.2
	Female	156	49.8
Age	50 ~ 59 years	179	57.2
	60 ~ 69 years	99	31.6
	70 ~ 79 years	29	9.3
	80 years and over	6	1.9
Monthly income (CNY)	Less than 3,000	44	14.1
	3,000 ~ 5,999	143	45.7
	6,000 ~ 11,999	94	30.0
	12,000 and above	32	10.2
Exercise intensity	Light (easy)	61	19.5
	Low (gentle)	164	52.4
	Moderate (active)	65	20.8
	High (short, intense)	15	4.8
	High (long, intense)	8	2.5
Exercise duration	Less than 10 min	12	3.8
	10 ~ 20 min	54	17.3
	21 ~ 30 min	72	23.0
	31 ~ 60 min	103	32.9
	More than 60 min	72	23.0
Exercise frequency	Once per month	17	5.4
	2 ~ 3 times per month	27	8.6
	1 ~ 2 times per week	75	24.0
	3 ~ 5 times per week	125	40.0
	Daily	69	22.0

### 3.3 Questionnaire design

This research scale encompasses the following concepts: recreational sports participation, resocialization, peer relationships, and well-dying perception. These scales were compiled from English-language literature and translated into the participants’ native language. To enhance the measurement tools’ adaptability and content validity, all scales underwent multiple reviews by two sports psychology professors and two physical education professors. They assessed the scales’ content and comprehensibility, proposing improvements to enhance clarity, readability, and content validity. Additionally, cultural sensitivity considerations were incorporated during the adaptation of the well-dying perception scale. Certain terminology underwent linguistic adaptation and semantic softening based on cultural context and the language comprehension characteristics of the elderly population. The measurement instruments in this study were based on established scales developed in prior literature. The first section covered demographic information, including gender, age, income, and exercise frequency. The second section comprised 31 items related to variables within the study’s conceptual model.

To measure recreational sports participation, this study adopted the scale developed by McIntyre and Pigram (1992), utilizing the questionnaire revised by Wu et al. (2020). This questionnaire comprises three subscales: Centrality (3 items), Attractiveness (3 items), and Self-Expression (3 items). It includes the following items: “I enjoy discussing sports with others,” “Participating in sports is important to me,” “I greatly enjoy participating in sports,” “When participating in sports, I feel free and true to myself,” “I am willing to let others know that I participate in sports,” etc. Responses were rated on a 5-point Likert scale (from “1 = Strongly Disagree” to “5 = Strongly Agree”).

To measure resocialization, this study adopted the scale developed by Kenyon and McPherson (1974), utilizing the questionnaire revised by Seo and Myung (2024). This questionnaire comprises three subscales: financial (3 items), physical (3 items), and social (3 items). It includes the following items: “I currently have no financial difficulties,” “I am highly active in social interactions,” “I greatly enjoy engaging in sports,” and “I am highly active in social interactions.” Responses were rated on a 5-point Likert scale (ranging from “1 = Strongly Disagree” to “5 = Strongly Agree”).

To measure peer relationships, this study employed a scale developed by Weiss and Smith (1999) and utilized a questionnaire revised by Pacewicz and Smith (2021). The questionnaire comprised four items assessing peer relationships, including the following statements: “I enjoy exercising with my friends,” “I like participating in sports with my friends,” “I feel well-matched when exercising with my friends,” and “I am willing to spend time exercising with my friends.” Responses were measured using a 5-point Likert scale (ranging from “1 = Strongly Disagree” to “5 = Strongly Agree”).

To measure well-dying perception, this study employed a scale developed by Schwartz et al. (2003) and utilized a questionnaire revised by Son (2020). This questionnaire contains questions related to end-of-life preferences (9 items), including the following statements: “I hope to pass away peacefully,” “I wish to die naturally without medical intervention,” “I hope to be free from pain before passing,” “I hope to maintain control over my body near the end of life,” “I hope to have the chance to say goodbye to my loved ones,” and other items. Responses were rated on a 5-point Likert scale (ranging from “1 = Strongly Disagree” to “5 = Strongly Agree”).

## 4 Results

### 4.1 Reliability and validity testing

To rigorously evaluate the psychometric quality of the measurement instruments used in this study, we conducted a series of reliability and validity assessments, with a primary focus on construct validity through CFA. The confirmatory factor analysis was employed to verify whether the observed variables adequately represented their respective latent constructs as specified in the theoretical model.

The CFA results are presented in Table 2. The overall model fit indices indicated a satisfactory fit between the proposed measurement model and the observed data: χ^2^/df = 2.145 (<3), IFI = 0.940, TLI = 0.931, CFI = 0.940, SRMR = 0.055, and RMSEA = 0.061. In general, an RMSEA and SRMR below 0.08 (and certainly below 0.10) reflect an acceptable fit, while IFI, TLI, and CFI values exceeding 0.90 are considered indicative of a well-fitting model (Hu and Bentler, 1999). The present results clearly meet these widely accepted thresholds, confirming the adequacy of the measurement model.

**Table 2:** Confirmatory factor analysis and reliability analysis.

Factor	Indicator	*β*	AVE	CR	*α*
Centrality	Q1: I enjoy discussing sports with others.	0.710	0.737	0.892	0.878
	Q2: Participating in sports is important to me.	0.942			
	Q3: Sports are an essential part of my life.	0.906			
Attraction	Q4: I greatly enjoy participating in sports.	0.908	0.823	0.933	0.932
	Q5: Sports are one of the most satisfying activities for me.	0.915			
	Q6: Participating in sports is enjoyable.	0.898			
Self-expression	Q7: Engaging in sports reflects my healthy lifestyle.	0.827	0.688	0.868	0.851
	Q8: When participating in sports, I feel true to myself.	0.914			
	Q9: I am willing to let others know that I participate in sports.	0.738			
Financial aspect	Q1: I currently have no financial difficulties.	0.839	0.763	0.906	0.904
	Q2: I have no outstanding debts.	0.854			
	Q3: I am not facing any financial problems at present.	0.924			
Physical aspect	Q4: I consider myself physically healthy.	0.856	0.632	0.836	0.825
	Q5: Compared to others of my age, I believe my physical strength is relatively good.	0.822			
	Q6: I greatly enjoy engaging in sports.	0.698			
Social aspect	Q7: I am highly active in social interactions.	0.742	0.626	0.834	0.829
	Q8: I am willing to treat my friends without hesitation.	0.783			
	Q9: I am still capable of contributing meaningfully to society.	0.841			
Peer Relationships	Q1: I enjoy exercising with my friends.	0.913	0.840	0.955	0.953
	Q2: I like participating in sports with my friends.	0.914			
	Q3: I feel well-matched when exercising with my friends.	0.913			
	Q4: I am willing to spend time exercising with my friends.	0.926			
Well-dying perception	Q1: I hope to pass away peacefully.	0.749	0.594	0.928	0.919
	Q2: I wish to die naturally without medical intervention.	0.583			
	Q3: I hope to be free from pain before passing.	0.876			
	Q4: I prefer not to experience a prolonged dying process.	0.815			
	Q5: I hope to maintain control over my body near the end of life.	0.808			
	Q6: I wish to communicate normally with others before passing.	0.879			
	Q7: I hope to remain conscious during my final moments.	0.671			
	Q8: I wish to have the opportunity to accomplish important tasks.	0.781			
	Q9: I hope to have the chance to say goodbye to my loved ones.	0.725			

Convergent validity was further assessed through the examination of standardized factor loadings, Average Variance Extracted (AVE), and Composite Reliability (CR). All standardized factor loadings were greater than 0.50, and most exceeded 0.70, indicating that each item made a substantial contribution to its respective latent construct (Hair et al., 2004). All AVE values surpassed the recommended threshold of 0.50, suggesting that, on average, more than half of the variance in the indicators was accounted for by their latent factor. Similarly, all CR values were greater than 0.70, signifying satisfactory internal consistency and reliability of the latent variables. Collectively, these results provide strong evidence for the convergent validity of the scales.

Reliability analysis was performed using Cronbach’s *α* coefficients for each latent variable. As shown in Table 2, all α values were above 0.80, which is well above the conventional cutoff of 0.70, indicating that the items within each construct consistently measure the same underlying concept. This level of internal consistency also supports the stability of the measurement over time and across different samples.

The final measurement model comprised eight latent constructs: centrality, attraction, self-expression, financial aspect, physical aspect, social aspect, peer relationships, and well-dying perception. Each construct was measured by between 3 and 9 items, all of which demonstrated strong psychometric properties. For example, the attraction dimension showed exceptionally high internal consistency (*α* = 0.932) and factor loadings above 0.89 for all items, reflecting a robust measurement of the intrinsic enjoyment of sports involvement. Similarly, the peer relationships scale exhibited both high reliability (*α* = 0.953) and excellent convergent validity (AVE = 0.840), highlighting the strong association between social bonds and exercise participation.

### 4.2 Correlation analysis

Pearson’s correlation analysis was conducted to examine the relationships among the study variables, with the results presented in Table 3. All correlation coefficients were below the threshold of 0.80, indicating the absence of multicollinearity and supporting the appropriateness of including these variables in subsequent regression analyses.

**Table 3:** Pearson’s correlation coefficients, descriptive statistics, and normality tests for study variables.

Variables	A	B	C	D	E	F	G	H
A	1							
B	0.699**	1						
C	0.559**	0.587**	1					
D	0.321**	0.261**	0.338**	1				
E	0.424**	0.521**	0.464**	0.436**	1			
F	0.387**	0.386**	0.457**	0.538**	0.489**	1		
G	0.520**	0.558**	0.565**	0.407**	0.579**	0.611**	1	
H	0.364**	0.293**	0.335**	0.239**	0.278**	0.366**	0.384**	1
Mean	4.276	4.279	4.246	3.941	4.111	4.044	4.247	4.602
SD	0.749	0.704	0.742	0.911	0.684	0.710	0.737	0.505
Skewness	−0.914	−0.543	−0.532	−0.617	−0.199	−0.308	−0.732	−1.152
Kurtosis	0.602	−0.723	−0.886	−0.356	−1.000	−0.503	−0.041	0.407

A = Centrality, B = Attraction, C = Self-expression, D = Financial aspect, E = Physical aspect, F = Social aspect, G = Peer relationships, H = Understanding well-dying; ^**^indicates *p* < 0.01.

To assess the normality of the data distribution, skewness and kurtosis statistics were examined for each variable. The skewness values ranged between −1.152 and −0.199, falling within the acceptable range of ±2, while kurtosis values ranged from −1.000 to 0.602, well within the recommended range of ±4 (Hong et al., 2003). These findings indicate that the data meet the assumption of univariate normality.

The correlation matrix shows that all study variables are significantly and positively related at the 0.01 level. For example, Centrality demonstrated a strong positive correlation with Attraction (*r* = 0.699, *p* < 0.01) and Peer Relationships (*r* = 0.520, *p* < 0.01), suggesting that individuals who regarded sports as central to their lives are also more likely to derive enjoyment from sports and engage in them with peers. Similarly, Peer Relationships showed substantial correlations with Self-expression (*r* = 0.565, *p* < 0.01) and Social Aspect (*r* = 0.611, *p* < 0.01), underscoring the interconnectedness of social engagement and personal expression through sports participation.

### 4.3 Hypothesis test

#### 4.3.1 Direct effects and mediating effects test

In order to validate the direct and indirect effects between involvement in recreational sports, resocialization, and well-dying perception, this study employed PROCESS Macro model 4 (Hayes, 2013). As shown in Table 4. The research findings indicate that the involvement in recreational sport had a significant positive effect on resocialization (*β* = 0.524, *p* < 0.001) and on well-dying perception (*β* = 0.221, p < 0.001). Resocialization had a significant positive effect on well-dying perception (*β* = 0.170, *p* < 0.01). These results support hypotheses H1, H2, and H3, confirming the prerequisite of resocialization as a mediating variable.

**Table 4:** Results of direct and indirect pathway analysis.

Independent variable	Dependent variable	Coeff	se	*t*
1. Gender	Resocialization	0.075	0.060	1.254
2. Age		0.044	0.041	1.094
3. Monthly income		0.204	0.035	5.878***
4. Exercise intensity		0.073	0.036	2.020*
5. Exercise duration		−0.008	0.031	−0.272
6. Exercise frequency		−0.006	0.030	−0.188
Recreational sport involvement		0.524	0.047	11.145***
*F* = 28.446***, *df* = 7/305, *R^2^* = 0.395				
1. Gender	Well-dying perception	0.135	0.055	2.461*
2. Age		−0.003	0. 037	−0.078
3. Monthly income		0.024	0.033	0.730
4. Exercise intensity		−0.082	0.033	−2.459*
5. Exercise duration		0.006	0.028	0.226
6. Exercise frequency		0.053	0.028	1.923
Recreational sport involvement		0.221	0.051	4.340***
Resocialization		0.170	0.052	3.258**
*F* = 11.476***, *df* = 8/304, *R^2^* = 0.232				

Path	Effect	Boot SE	BootLLCI	bootULCI
Recreational sport involvement → Resocialization → Understanding well-dying	0.089	0.032	0.031	0.155

*indicates *p* < 0.05, **indicates *p* < 0.01, ***indicates *p* < 0.001.

In addition, involvement in recreational sport had a significant positive effect on resocialization (*β* = 0.524, *p* < 0.001) and on well-dying perception (*β* = 0.221, *p* < 0.001). Resocialization had a significant positive effect on well-dying perception (*β* = 0.170, *p* < 0.01). These results support hypotheses H1, H2, and H3, confirming the prerequisite of resocialization as a mediating variable.

In PROCESS Macro model 4, mediating effects were tested using Bootstrap (Bootstrapping: 10,000, 95% CI). The results indicated that the mediating effect of resocialization between involvement in recreational sport and well-dying perception (*β* = 0.089, CI = 0.031–0.155) had a 95% confidence interval excluding zero—indicating a significant mediating effect. These findings suggest that older adults’ recreational sport involvement not only positively affects their well-dying perception directly, but also indirectly through resocialization. The results support hypothesis H4.

#### 4.3.2 The moderated mediating effect of peer relationships

**Figure 2:**
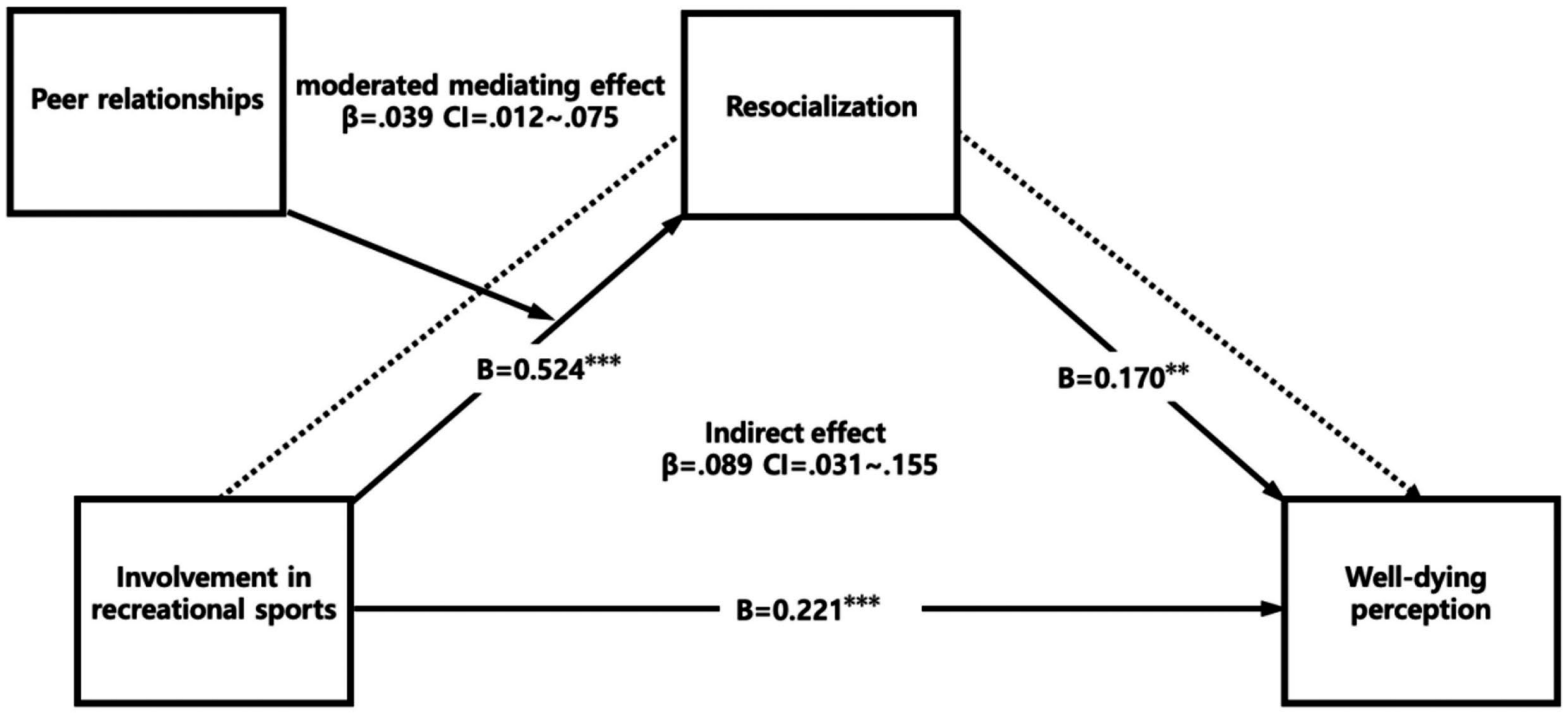
Path results between involvement in recreational sports, resocialization, peer relationships, and well-dying perception. The dashed line indicates a mediating path between resocialization and both recreational sports participation and well-dying perception. All paths are statistically significant.

We used Hayes’ PROCESS (model 7) to test the moderated mediating effect. The results are presented in Table 5, and the path model is illustrated in Figure 2.

**Table 5:** The moderating role of peer relationships.

Dependent variable	Independent variables	Coeff	se	*t*
Resocialization	1. Gender	0.094	0.052	1.807
	2. Age	0.062	0.035	1.751
	3. Monthly income	0.183	0.030	6.057***
	4. Exercise intensity	0.066	0.032	2.076*
	5. Exercise duration	−0.036	0.027	−1.319
	6. Exercise frequency	0.005	0.026	0.201
	Recreational sport involvement	0.275	0.051	5.441***
	Peer relationships	0.420	0.044	9.477***
	Recreational sport involvement * Peer relationships	0.232	0.048	4.850***
*F* = 40.443***, *df* = 9/303, *R^2^* = 0.546				
Well-dying perception	1. Gender	0.135	0.055	2.461*
	2. Age	−0.003	0.037	−0.078
	3. Monthly income	0.024	0.033	0.730
	4. Exercise intensity	−0.082	0.033	−2.459*
	5. Exercise duration	0.006	0.028	0.226
	6. Exercise frequency	0.053	0.028	1.923
	Recreational sport involvement	0.221	0.051	4.340***
	resocialization	0.170	0.052	3.258**
*F* = 11.476***, *df* = 8/304, *R^2^* = 0.232				

* indicates *p* < 0.05, ** indicates *p* < 0.01, ***indicates *p* < 0.001.

As shown in Table 5, involvement in recreational sports (*β* = 0.275, *p* < 0.001) and peer relationships (*β* = 0.420, *p* < 0.001) had significant positive effects on resocialization. Resocialization (*β* = 0.170, *p* < 0.01) and involvement in recreational activity (*β* = 0.221, *p* < 0.001) also had significant effects on well-dying perception. Furthermore, peer relationships significantly moderated the relationship between involvement in recreational activity and resocialization (*β* = 0.232, *p* < 0.001).

The moderated mediation index further confirmed the presence of a moderated mediation effect of peer relationships. As shown in Table 6, the 95% confidence interval for the dependent variable, well-dying perception, was (0.012, 0.075), excluding zero, indicating a significant moderated mediation effect. The effect index was 0.039. Finally, the 95% confidence intervals confirmed that peer relationships, as a moderating and mediating variable, had a significant effect in the average group (M) and the high group (M + 1SD). In conclusion, compared with older adults with average peer relationships, those with stronger peer relationships showed a greater indirect effect of involvement in recreational sports on well-dying perception through resocialization.

**Table 6:** Index of moderated mediating and the moderating effect of peer relationships in low, average, and high groups.

Level of peer relationships	Index	Boot SE	BootLLCI	BootULCI
Index of moderated mediation	0.039	0.016	0.012	0.075
Low (−0.737)	0.018	0.013	−0.002	0.047
Average (0)	0.047	0.019	0.015	0.091
High (0.737)	0.076	0.030	0.025	0.142

## 5 Discussion

### 5.1 Theoretical contributions

First, focusing on older adults’ well-dying perception, this study examined the potential relationships among physical functioning, active social participation, and cognition within the framework of successful aging theory. The findings indicate that involvement in recreational sports may connect these three core elements in everyday life. In other words, the role of involvement in recreational sports among older adults is not limited to improving physical health; it also provides a pathway through which they may reintegrate into society (Oude Groeniger et al., 2019). At the same time, older adults may gradually develop a more positive well-dying perception through active and sustained involvement in recreational sports. Compared with previous research, this finding offers a new perspective. Earlier studies have suggested that older adults tend to reflect on how to face death primarily when confronted with illness or end-of-life experiences (Clarke et al., 2012). However, the present study suggests that older adults’ well-dying perception does not necessarily emerge only from events occurring at the end of life; rather, it may gradually develop through sustained involvement in recreational sports (Howes, 2016). One possible explanation is that, during sport participation, older adults accomplish goals and experience a sense of control over their bodies, which may help them recognize that later life is not defined solely by decline but can still involve the maintenance of physical capability. These experiences may encourage older adults to perceive death as a natural stage in the life course and to develop a more positive, well-dying perception.

Second, this study verified the internal mechanism through which active social participation links physical functioning and cognition. The results indicate that active social participation is a key factor connecting the body and cognition. Although involvement in recreational sports itself has health-promoting benefits, its effect on well-dying perception may not arise solely from physical improvement; rather, it may operate through the process of resocialization. These findings are consistent with previous research showing that active social participation can reduce death anxiety (Zhou et al., 2024) and buffer various age-related adverse outcomes (Mendes de Leon et al., 2003). According to Kawachi and Berkman (2001), socially engaged older adults are more likely to experience happiness, whereas those with lower levels of social participation are more likely to experience anxiety (Chiao et al., 2011). Successful aging theory also emphasizes that active social participation is an essential component of successful aging (Rowe and Kahn, 1997). After retirement, older adults often face a reduction in social roles and a decline in social functioning (Cao et al., 2025). In this context, involvement in recreational sports provides a new social platform that enables older adults to remain connected with peers, friends, and the broader society while also satisfying their social needs. Through this process, resocialization promotes social connectedness, social support, information exchange, and a sense of identity among older adults, ultimately playing an important role in strengthening their well-dying perception.

Third, this study examined the boundary role of peer relationships, thereby further clarifying the conditions under which older adults’ well-dying perception is more likely to develop. The findings show that, compared with older adults with average levels of peer relationships, those with stronger peer relationships are more likely to experience enhanced resocialization during involvement in recreational sports, which ultimately contributes to improved well-dying perception. One possible explanation is that participation in sports alone may not be sufficient to generate meaningful social integration; the quality of the social environment in which participation occurs is equally important. Peer relationships can provide older adults with emotional support, encouragement to participate, and sustained interaction (Pierre et al., 2015). When older adults engage in sport with peers who share similar life experiences and aging-related circumstances, involvement in recreational sports is no longer merely an individualized form of physical activity. Instead, it becomes a platform for mutual recognition and social connection. Such peer support may strengthen the process of resocialization and enhance the positive effect of involvement in recreational sports on well-dying perception. As previous research has shown, peer relationships are an important factor influencing social participation (Fredricks and Simpkins, 2013). In addition, positive peer relationships can increase individuals’ willingness to continue participating in recreational sports and improve their physical and psychological health (Chang et al., 2014). These findings suggest that peer relationships, as an important social resource, may amplify the positive effects of involvement in recreational sports on older adults’ physical and mental health by strengthening social support and sense of belonging.

Existing research has not sufficiently examined the potential mechanisms through which involvement in recreational sports influences well-dying perception, particularly with respect to the joint roles of social participation and social relationships. This study innovatively extends the concept of well-dying perception beyond the domains of medical care and end-of-life care by situating it within the broader process of aging. At the same time, this study enriches successful aging theory by demonstrating that its three core dimensions are not independent outcomes but are closely interconnected. In addition, by incorporating both social participation and social relationship factors, this study systematically examines the mediating role of resocialization in the relationship between involvement in recreational sports and well-dying perception, as well as the moderated mediating role of peer relationships. Therefore, this study advances understanding of the mechanisms underlying the formation of a well-dying perception among older adults and provides valuable insights into how successful aging and holistic well-being may be promoted in later life.

### 5.2 Practical implications

Governments and communities should pay more attention to the promotion and popularization of recreational sports for the elderly. Recreational sports should be incorporated into the policy system of successful aging and end-of-life care. Priority should be given to the promotion of sports that are suitable for the physical condition and psychological needs of the elderly, such as yoga, meditation, and dancing, to name just a few. These sports not only enhance physical function, but also have the effect of relaxing the body. These exercises not only enhance physical functioning but also have the effect of relaxing emotions and increasing inner awareness (Rojas, 2025). More importantly, these exercises can be used as a vehicle for well-dying perception by integrating them with life review or end-of-life preparation to guide older adults to think about the meaning of life.

Additionally, the change of identity role and the weakening of social functioning of older adults after retirement may lead to loneliness and death anxiety (Froidevaux et al., 2022). Therefore, communities can organize sports competitions or festivals for older adults, encouraging them to participate not only as participants, but also as referees, moderators, and volunteers. This kind of participation not only gives older adults new social responsibilities but also stimulates their sense of self-worth and social belonging (Wood and Dashper, 2022). In addition, the ceremonial and public nature of these activities helps to shape the social image of older people as active participants in life, thereby enhancing their psychological resilience in the face of aging and the end of life.

Moreover, to enhance the overall well-being of the elderly, we should not only focus on the frequency and intensity of exercise, but also consciously encourage the elderly to participate in group exercise with social interaction characteristics. Such exercises help to strengthen peer relationships, bring a sense of social belongingness and psychological support to older people, and promote well-being in old age.

## 6 Limitations and future research directions

This study is not without limitations. First, the sample size of this study is limited to older adults in China, which may differ from those in other regions. Future research could expand the sample size to include older adults from different countries and regions to explore the effects of differences in cultural, social policy, and living environment on the relationship between Recreational sport involvement and well-dying perception. Second, the cross-sectional design of this study makes it difficult to determine the chronological order of the associations between involvement in recreational sports, resocialization, peer relationships, and well-dying perception among older adults. Future studies may adopt longitudinal research designs to further investigate the causal relationships among these variables. Finally, due to the sensitive nature of well-dying perception, the questionnaire survey may be affected. Future research could incorporate in-depth interviews to further explore the perception of end-of-life among older people.

## 7 Conclusion

This study validated the relationship between recreational sport involvement, resocialization and well-dying perception among older adults. The moderated mediating effect of peer relationships was analyzed. The findings showed that recreational sport involvement and resocialization affected well-dying perception, and resocialization had a positive mediating effect between involvement in recreational sport and perception of well-dying. This suggests that older adults’ resocialization through exercise may help them to face the end of life more positively. Finally, peer relationships had a moderated mediating effect on the pathway from recreational sport involvement and resocialization to understanding well-dying, suggesting that peer relationships can modulate the strength of the effect of recreational sport involvement on resocialization.

Based on the results of this study, when promoting the health of older adults, we should not only focus on the frequency and intensity of exercise, but also emphasize the social interactions and emotional connections established through participation in exercise. We should consciously create more social platforms and communication opportunities for older adults to help them find a sense of social belonging after retirement. In addition, more attention should be paid to strengthening geriatric education, especially the correct guidance on the knowledge and attitude towards death, which should be included in geriatric education and public health promotion programs.

## Data Availability

The raw data supporting the conclusions of this article will be made available by the authors, without undue reservation.
